# Osteocalcin attenuates high fat diet-induced impairment of endothelium-dependent relaxation through Akt/eNOS-dependent pathway

**DOI:** 10.1186/1475-2840-13-74

**Published:** 2014-04-07

**Authors:** Jianxin Dou, Huating Li, Xiaojing Ma, Mingliang Zhang, Qichen Fang, Meiyun Nie, Yuqian Bao, Weiping Jia

**Affiliations:** 1 Department of Endocrinology and Metabolism, Shanghai Jiao Tong University Affiliated Sixth People’s Hospital, Shanghai Clinical Center for Diabetes, Shanghai Key Laboratory of Diabetes Mellitus, Shanghai Diabetes Institute, 600 Yishan Road, Shanghai 200233, China

**Keywords:** ApoE knockout mice, Osteocalcin, Endothelium-dependent relaxation, PI3K/Akt/eNOS signaling pathway, Metabolic syndrome, Atherosclerosis

## Abstract

**Background:**

Recent studies have demonstrated a protective effect of osteocalcin (OCN) on glucose homeostasis and metabolic syndrome. However, its role in vascular function remains unknown. This study investigated the contribution of OCN to the pathogenesis of endothelial dysfunction in the thoracic aorta of apolipoprotein E-deficient (ApoE-KO) mice.

**Methods:**

Eight-week-old ApoE–KO mice were given chow or high fat diet (HFD) for 12 weeks with or without daily intraperitoneal injection of OCN. Intraperitoneal glucose tolerance test (IPGTT), insulin tolerance test (ITT),measurement of serum lipid profiles and blood pressure were carried out. Endothelium-dependent relaxation (EDR) was measured by wire myography. Human umbilical vein endothelial cells (HUVECs) were used to study the role of OCN on eNOS levels *in vitro*. PI3K inhibitor (LY294002) and Akt inhibitor V were used *ex-vivo* to determine whether PI3K/Akt/eNOS contributes to the beneficial effect of OCN for the vascular or not.

**Results:**

Daily injections of OCN can significantly improve lipid metabolism, glucose tolerance and insulin sensitivity in ApoE-KO mice. In ApoE-KO mice fed with HFD, the OCN-treated mice displayed an improved acetylcholine-stimulated EDR compared to the vehicle-treated group. In addition, compared to vehicle-treated HUVECs, OCN-treated HUVECs displayed increased activation of the Akt-eNOS signaling pathway, as evidenced by significantly higher levels of phosphorylated Akt and eNOS. Furthermore, a similar beneficial effect of OCN on thoracic aorta was observed using *ex vivo* organ culture of isolated mouse aortic segment. However, this effect was attenuated upon co-incubation with PI3K inhibitor or Akt inhibitor V.

**Conclusions:**

Our study demonstrates that OCN has an endothelial-protective effect in atherosclerosis through mediating the PI3K/Akt/eNOS signaling pathway.

## Background

Osteocalcin (OCN), the most abundant noncollagenous protein in bone, is an important clinical marker of bone formation and bone turnover. Studies of OCN levels in patients with metabolic dysfunctions have revealed a close correlation with perturbed OCN levels [1,2]. Loss- and gain-of-function experiments in mice have identified systemic roles of OCN in glucose, lipid and energy metabolism, whereby the protein ultimately promotes pancreatic insulin production and secretion [3,4]. Similarly, several recent studies in humans have demonstrated independent inverse correlations between serum OCN level and plasma glucose concentration, dyslipidemia, and metabolic syndrome; each of these clinical parameters are related to atherosclerosis, and are, therefore, risk factors for cardiovascular disease [5-8]. In addition, enhanced levels of OCN have been detected proximal to calcified plaques of human and rodent vascularization [9-11]. Taken together, these results suggest a close relationship between OCN and vascular disease.

The relationship between OCN and vascular disease still remains controversial. Results of a clinical trial which investigated the relationship between OCN and arterial stiffness showed that serum OCN level was inversely associated with brachial-ankle pulse-wave velocity in patients with T2DM [12]. Previous research by our group indicated that an inverse association may exist between serum OCN level and the severity of coronary atherosclerosis in Chinese men and with carotid intima-media thickness in Chinese postmenopausal women [7,13]. Another clinical study conducted by Li S, et al. also confirmed the results mentioned above [14]. Moreover, the potential protective effect of OCN against development of vascular disease has been consistently evidenced in both *in vitro* experiments and animal studies [15]. Nevertheless, a study by Atsushi A, et al. concluded a negative result that they thought serum osteocalcin did not show any relationship with vascular calcification [16].

Endothelial dysfunction is an early step in the development of atherosclerosis. It contributes to the initiation and early progression of atherosclerosis. In addition, endothelial dysfunction, as an independent predictor of cardiovascular events, has been consistently associated with obesity and the metabolic syndrome in a complex interplay with insulin resistance. However, the specific contribution of OCN to vascular dysfunction remains largely unknown. Thus, the current study was designed to investigate the role and molecular mechanism of OCN in endothelium-dependent relaxation (EDR) by using a mouse model of atherosclerosis and cultured human endothelial cells.

## Materials and methods

### Antibodies and chemicals

Rabbit anti-mouse phosphorylated (P)-Akt (Ser473) (1:1000 dilution), total-Akt (1:1000), P-endothelial nitric oxide synthase (eNOS; Ser1177) (1:1000), total-eNOS (1:1000), P-PI3K (1:1000), total-PI3K (1:1000) and GAPDH (1:2500) were purchased from Cell Signaling Technology (Danvers, MA, USA). OCN was obtained from Thermo Fisher Scientific, Inc. (Waltham, MA, USA). Phenylephrine (PE), acetylcholine (Ach), *N*^G^-nitro-L-arginine methyl ester (L-NAME), sodium nitroprusside (SNP), phosphatidylinositide (PI)3-kinase inhibitor LY294002, and protein kinase B (Akt) inhibitor V were purchased from Sigma-Aldrich (St. Louis, MO, USA) and dissolved in recommended solvents. Dulbecco’s modified Eagle’s medium (DMEM), fetal bovine serum and antibiotics were obtained from Gibico (Gaithersburg, MD, USA). Insulin ELISA kit was offered by Antibody and Immunoassay Service Center in Hong Kong University. TNF-α, IL-1α, IL-12 p70 and IL-12 p40 ELISA kits were purchased from R&D Systems (Minneapolis, Minnesota, USA).

### Cell culture

Human umbilical vein endothelial cells (HUVECs) were isolated from fresh human umbilical veins (neonate cords donated by the Shanghai Jiao Tong University affiliated Sixth People’s Hospital, China) using 0.125% trypsase and cultured in endothelial basal medium (EBM-2; cc-3202, Lonza Group, Ltd., Basel, Switzerland) supplemented with 2% fetal bovine serum and various endothelial-cell growth factors at 37°C in a 95% O_2_/5% CO_2_ humidified incubator. Culture passages 2 through 4 were used for the experiments.

### Animals and in vivo study design

Male ApoE-KO mice established on a C57BL/6 genetic background (7 weeks-old; 18 – 20 g) were purchased from the Medical Research Center of Peking University (Beijing, China). The mice were housed in filter-topped cages under pathogen-free conditions with a 12-hour light/dark cycle (darkness from 7:30 p.m. to 7:30 a.m.), 23 ± 1°C constant temperature, 55–60 humidity, and *ad libitum* access to standard laboratory food and water. All mice were acclimatized to the laboratory environment for one week prior to experimentation. All experimental protocols complied with institutional guidelines for the humane treatment of laboratory animals. The protocol was approved by the ethics committee of Shanghai Jiao Tong University Affiliated Sixth People’s Hospital, following the priciples of the Declaration of Helsinki.

At 8 weeks-old, the mice were randomly selected for receipt of chow diet or high fat diet (HFD; 42% fat and 0.15% cholesterol [TD88137; Harlan Teklad, Madison, WI, USA]) for 12 weeks, with or without daily (4:00 pm) intraperitoneal (i.p.) injection of 10 μL/g OCN (at 3 ng/μL freshly diluted in normal saline (0.9% NaCl; vehicle)). Body weight was measured weekly by electronic balance.

### Intraperitoneal glucose tolerance test (GTT) and Insulin tolerance test (ITT)

After overnight fasting and i.p. injection of glucose (1.5 g/kg body weight), tail vein blood sampling was carried out at the indicated time points. Glucose and insulin levels were measured by glucometer (ACCU-CHEK; Roche Diagnostics Inc., Indianapolis, IN, USA) and enzyme-linked immunosorbent assay, respectively. The mouse insulin ELISA kit had intra- and interassay coefficients of variation of 10% and 10%, respectively.

After 6 h of fasting and i.p. injection of insulin (0.5 U/kg of Humulin; Eli Lilly, Inc., Indianapolis, IN, USA), tail vein blood sampling was carried out at the indicated time points. Blood glucose was measured as above.

### Determination of blood parameters

All collected blood samples were stored at −80°C until analysis. Fasting blood glucose (FBG) measurements were represented by the blood draws taken at the appropriate 6 and 12 h fasting time points, prior to glucose or insulin injection. After 12 weeks of experimental intervention (20-week-old animals), serum lipid profiles were measured enzymatically using an autoanalyser (7600–120 Automatic Analyser, Hitachi Inc., Tokyo, Japan) and included detection of triglycerides (TG), total cholesterol (TC), high-density lipoprotein cholesterol (HDL-C), and low-density lipoprotein cholesterol (LDL-C). Serum levels of TNF-α, IL-1α, IL-12 p70 and IL-12 p40 were measured by ELISA.

### Measurement of blood pressure

Blood pressure measurements were made on 20-week-old animals after the experimental intervention. Systolic, mean, and diastolic blood pressures (BPs) were measured by a programmable sphygmomanometer (BP-98A; Softron Co Ltd, Tokyo, Japan) using a tail-cuff method described previously [17].

### Measurement of vascular reactivity in isolated ApoE-KO aortic strips

All mice were sacrificed after the 12th week of experimental intervention, and the descending thoracic aorta (2-mm long) was excised and immediately submerged in oxygenated ice-cold Krebs solution (in mmol/L: 130 NaCl, 4.7 KCl, 1.6 CaCl_2_, 1.17 MgSO_4_ · 7 H_2_O, 14.9 NaHCO_3_, 1.18 KH_2_PO_4_, 0.026 EDTA and 5.5 glucose). Changes in isometric tone of the aortic strips were recorded by wire myography (Danish Myo Technology, Aarhus, Denmark). The strips were stretched to an optimal baseline tension of 5 mN and allowed to equilibrate for 60 min prior to experimentation. Subsequently, the strips were contracted with 60 mmol/L KCl and rinsed several times in Krebs solution. Phenylephrine (PE; 10^−5^ M) was added to produce a steady contraction. The EDR triggered upon addition of increasing concentrations of acetylcholine (ACh; 10^−9^ M to 10^−5^ M) were recorded.

In a separate set of experiments, aortic strips were incubated with *N*^*G*^-nitro-L-arginine methyl ester (L-NAME; 10^−4^ M) for 30 min, followed by precontraction and determination of EDR. Relaxation response to sodium nitroprusside (SNP; 1 nM to 1 μM) was also investigated to test the responsiveness of the vascular smooth muscle cells (VSMCs) to exogenous nitric oxide (NO). The recorded relaxation values were plotted as percentages of the contraction values induced by PE.

### Western blot analysis

Equal amounts of total protein extracts from cells and tissues were separated by sodium dodecyl sulfate-polyacrylamide gel electrophoresis (SDS-PAGE) and probed with different primary antibodies as specified in each figure legend. The specific signals were amplified by addition of horseradish peroxidase-conjugated secondary antibodies and visualized using an enhanced chemiluminescence system (Amersham). The intensity of the protein bands was quantified using Quantity One software (Bio-Rad, Hercules, CA, USA).

### Ex vivo culture of mouse thoracic aortic strips

In another experiment, strips of the mouse descending thoracic aortic tissues (2 mm in length) from vehicle-treated mice fed with HFD were dissected in sterile phosphate buffered saline and submerged in DMEM (with 584 mg/L L-glutamine, 4500 mg/L D-glucose,110 mg/L sodium Pyruvate, pyridoxine hydrochloride), supplemented with 10% fetal bovine serum plus 100 IU/mL penicillin and 100 μg/mL streptomycin. OCN (58 ng/ml), LY294002 (PI3K inhibitor, 10 μmol/L), or Akt inhibitor V (API-2/triciribine, 5 μmol/L) was added individually to the culture medium for 24 h of incubation under oxygenated condition (95% O_2_,5% CO_2_) at 37°C. After transferring the strips to a chamber filled with fresh Krebs solution, changes in isometric force were measured by wire myography [18].

### Statistical analysis

All data are expressed as the mean ± standard error of the mean (SEM) and were processed with SPSS version 16.0 software (SPSS Inc., Chicago, IL, USA). The significance (*P* < 0.05) of intergroup differences was assessed by two-tailed paired Student’s *t*-test or one-way ANOVA, as appropriate.

## Results

### Daily injections of OCN improved lipid metabolism, glucose tolerance, insulin sensitivity and alleviated inflammation in ApoE-KO mice

**Figure 1:**
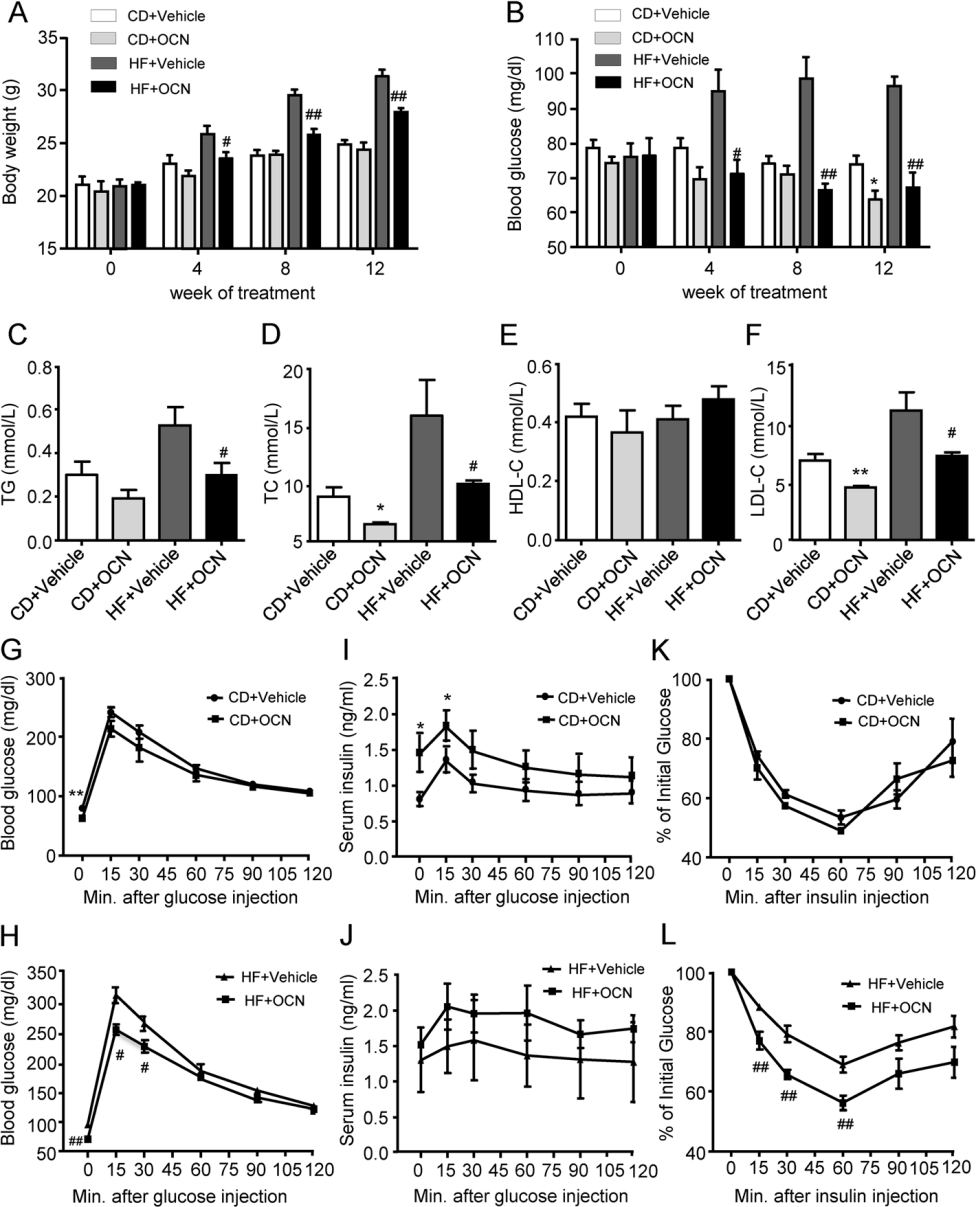
**Daily injections of OCN improve glucose tolerance and lipid metabolism in ApoE-KO mice.** Analyses were performed in mice injected daily with the indicated doses of OCN or with vehicle for the indicated time. During 12 weeks of experimental intervention, body weight **(A)**, FBG **(B)** was measured. After 12 weeks of experimental intervention, measurements were taken of serum lipid levels **(C-F)**, glucose during GTT **(G-H)**, insulin during GTT **(I-J)**, and glucose by ITT **(K-L)**. Values represent mean ± SEM, 6 to 8 mice per group were analyzed. *P < 0.05, **P < 0.01, OCN-treated mice vs. vehicle-treated mice in chow diet group; ^#^*P <* 0.05, ^##^*P <* 0.01, OCN-treated mice vs. vehicle-treated mice in HFD group. HF, high fat diet; CD, chow diet; OCN, osteocalcin; FBG, fasting blood glucose.

**Figure 2:**
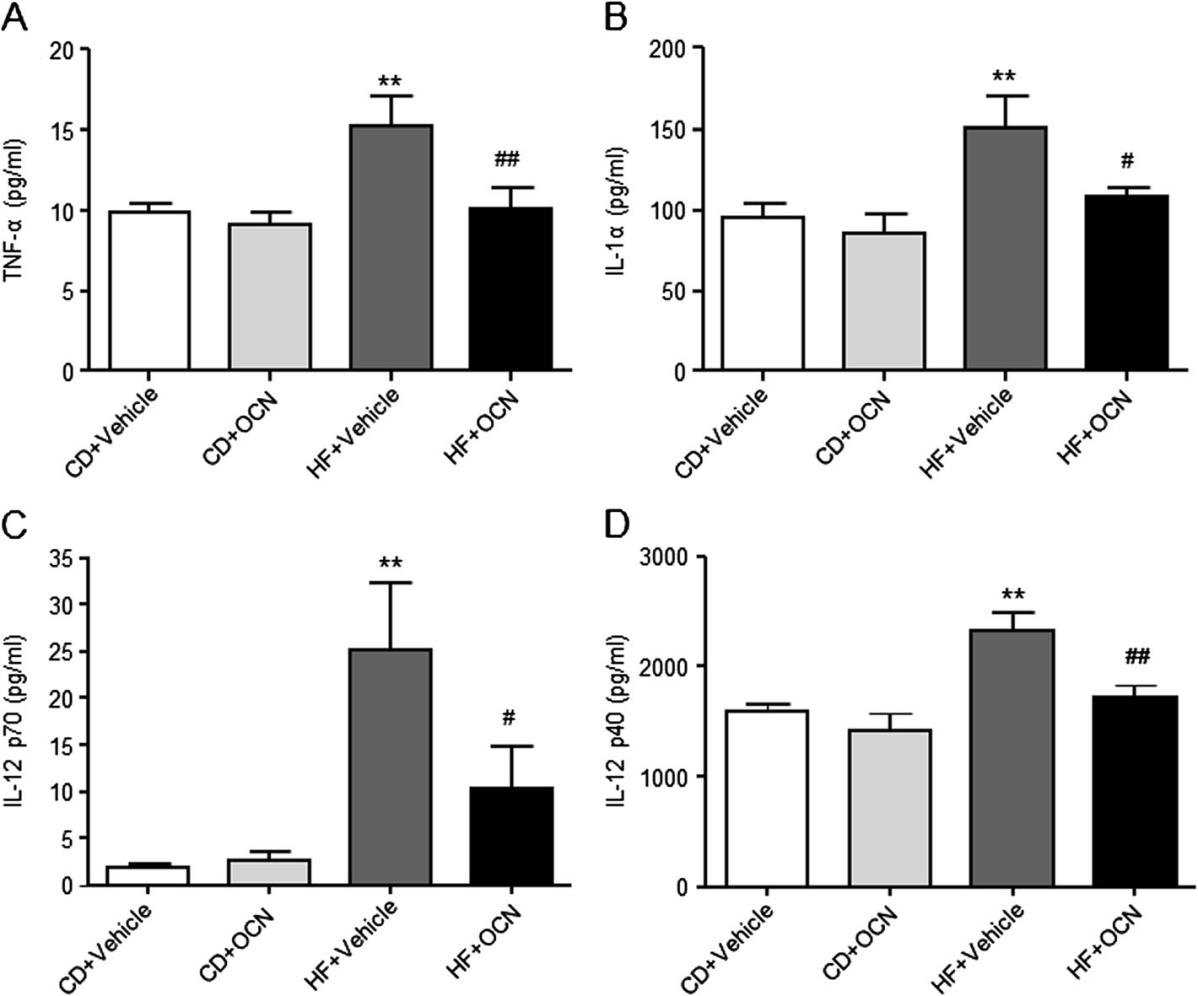
**Serum levels of atherogenic inflammatory factors decreased in OCN-treated compared with vehicle-treated ApoE-KO mice. (A)** TNF-α **(B)** IL-1α **(C)** IL-12 p70 **(D)** IL-12 p40. Data were shown as mean ± SEM, 6 to 8 mice per group were analyzed. **P < 0.01 vehicle-treated mice fed with HFD vs. vehicle-treated mice fed with chow diet; ^#^*P <* 0.05, ^##^*P <* 0.01 OCN-treated mice fed with HFD vs. vehicle-treated mice fed with HFD. HF, high fat diet; CD, chow diet; OCN, osteocalcin.

At baseline, there was no significant difference in body weight or FBG levels among the four groups. After 12 weeks of experimental intervention, the OCN-treated mice fed with HFD showed a significantly lower body weight than the vehicle-treated mice fed with HFD (27.9 ± 0.39 vs. 31.33 ± 0.60 g, *P* < 0.01) (Figure 1A). Compared with the vehicle-treated mice fed with chow diet, the OCN-treated mice fed with chow diet had significantly lower FBG level after 12 weeks of experimental intervention (*P* = 0.021). Furthermore, the decrease of FBG was more obvious in the OCN-treated mice fed with HFD (*P <* 0.01) (Figure 1B). The effect of OCN on lipid profiles was also assessed. In mice fed with chow diet, serum levels of TC and LDL-C were significantly lower in the OCN-treated mice compared with the vehicle-treated mice. In mice fed with HFD, serum levels of TC, TG and LDL-C significantly decreased in OCN-treated mice compared with the vehicle-treated mice (all *P* < 0.05) (Figure 1C–F). IPGTT and ITT were conducted to determine if intermittent administration of OCN can affect glucose metabolism in ApoE-KO mice. In mice fed with chow diet, the OCN-treated mice only showed an improving tendency of both glucose tolerance and insulin sensitivity (vs. vehicle-treated mice) with no significant difference detected among the measured values in these two groups (Figure 1G, K). However, in mice fed with HFD, glucose tolerance and insulin sensitivity of OCN-treated mice significantly improved, compared with the vehicle-treated mice (Figure 1H, L). In addition, insulin secretion also improved in both groups of OCN-treated mice (Figure 1I, J). These results suggest that daily injections of OCN is likely to improve glucose and lipid metabolism,all of which are the risk factors of cardiovascular disease. Additionally, serum levels of atherogenic inflammatory factors were also measured. Compared with the mice fed with chow diet, serum levels of TNF-α, IL-1α, IL-12 p70 and IL-12 p40 significantly increased in mice fed with HFD. Furthermore, serum levels of these inflammatory factors significantly decreased after OCN treatment in mice fed with HFD (Figure 2).

### OCN reduces diastolic BP and the mean blood pressure in ApoE-KO mice

**Figure 3:**
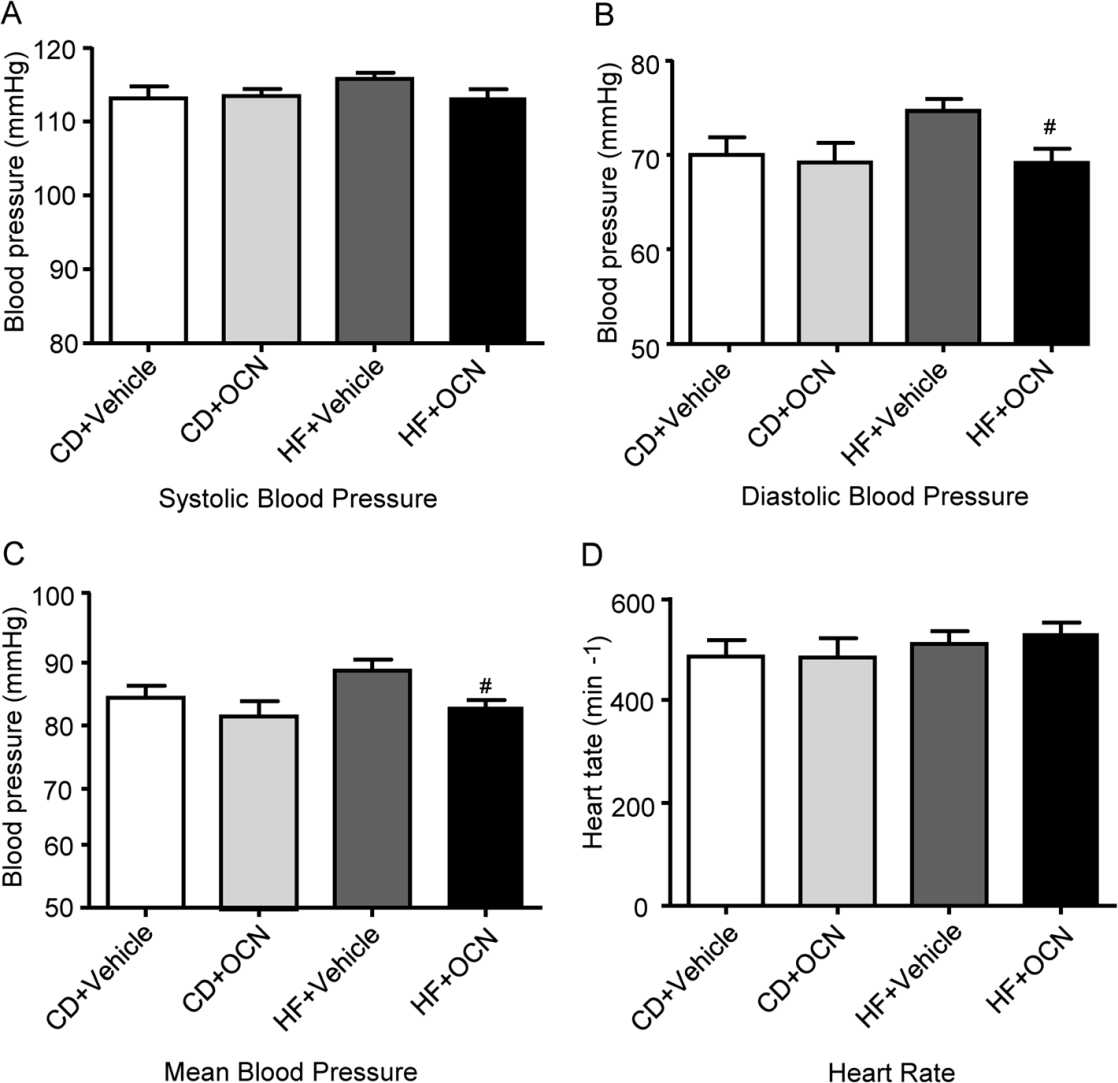
**Blood pressure measured in OCN-treated and vehicle-treated ApoE-KO mice. (A)** Systolic blood pressure. **(B)** Diastolic blood pressure. **(C)** Mean blood pressure. **(D)** Heart rate. Values represent mean ± SEM, 6 to 8 mice per group were analyzed. ^#^*P <* 0.05, OCN-treated mice fed with HFD vs. vehicle-treated mice fed with HFD. HF, high fat diet; CD, chow diet; OCN, osteocalcin.

Blood pressure was measured after 12 weeks of experimental intervention. No significant differences were detected in systolic blood pressure (SBP) when comparing among all these four groups (Figure 3A). In mice fed with chow diet, the OCN-treated mice only showed a decreasing tendency in mean blood pressure without reaching the level of significance. However, the OCN intervention did lead to a significant reduction of diastolic BP (*P* < 0.05), as well as mean blood pressure in mice fed with HFD. (*P* < 0.05) (Figure 3B-C). In addition, no significant differences were found in the comparison of heart rate among four groups (Figure 3D).

### OCN attenuates impairment of EDR in ApoE-KO aortic strips

**Figure 4:**
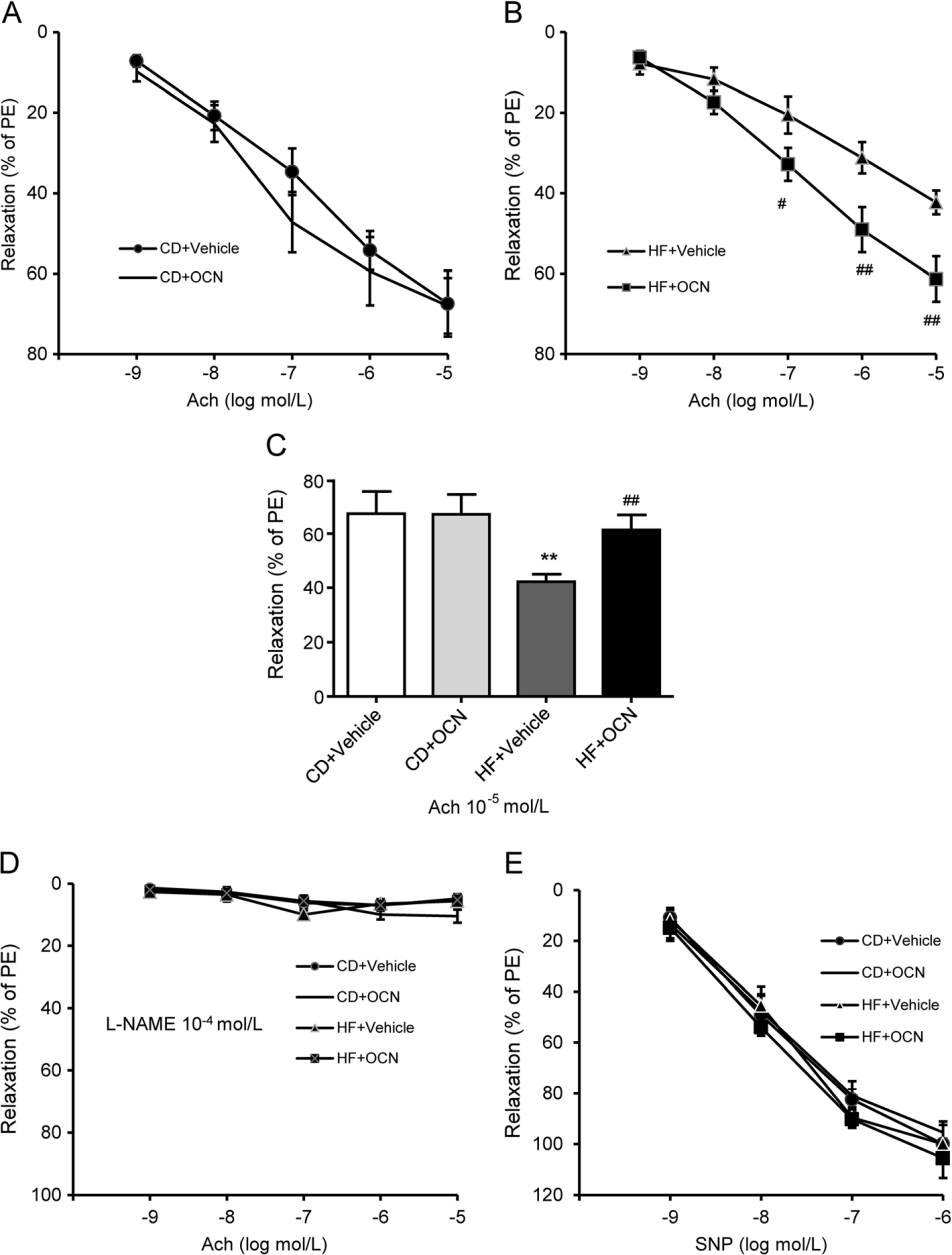
**Effect of OCN on EDR in descending thoracic aortic strips of ApoE-KO mice. (A–B)** ACh-mediated EDR. **(C)** Maximal relaxation values. **(D)** ACh-induced EDR in the presence of L-NAME. **(E)** Endothelium-independent relaxations to SNP. Values represent mean ± SEM, 6 to 8 mice per group were analyzed. **P < 0.01 vehicle-treated mice fed with HFD vs. vehicle-treated mice fed with chow diet; ^#^*P <* 0.05, ^##^*P <* 0.01 OCN-treated mice vs. vehicle-treated mice in HFD group. HF, high fat diet; CD, chow diet; EDR, endothelium-dependent relaxation; OCN, osteocalcin.

Following the sacrifice of the ApoE-KO mice, the aortic arches were taken and any adventitial fat was dissected away before cutting the aortic arch longitudinally. The aortic arches were stained with Oil-Red-O. All of the ApoE-KO mice have developed atherosclerosis after the 12th week of experimental intervention (Additional file 1: Figure S1). The descending thoracic aorta was used to measure the vascular reactivity. The ACh-stimulated EDR of aortic strips was lower in the vehicle-treated mice fed with HFD than vehicle-treated mice fed with chow diet (maximal relaxation (Emax) = 42.30 ± 2.95 vs. 67.43 ± 8.20%, *P* < 0.01). The ACh-stimulated EDR was unaltered in the OCN-treated mice fed with chow diet. However, in mice fed with HFD, the OCN treatment induced a significant increase in ACh-stimulated EDR (Emax = 42.30 ± 2.95 vs. 61.34 ± 5.64%, *P* < 0.01) (Figure 4A-C). When incubated with L-NAME, a NO-synthase inhibitor, the ACh-induced vasodilatory response was predominantly abolished in all groups (Figure 4D). Endothelium-independent vasodilation was studied through the addition of SNP, a NO donor that bypasses endogenous endothelial-cell NO production. Since the level of SNP-stimulated NO-dependent relaxation of VSMCs was similar among all four groups, it appeared that neither diet nor OCN treatment affected VSMC responsiveness to NO in ApoE-KO mice (Figure 4E). These results suggest that OCN treatment improved EDR in aortae from ApoE-KO mice fed with HFD, without affecting endothelium-independent relaxations to SNP.

### OCN increases eNOS phosphorylation in ApoE-KO aortic strips

**Figure 5:**
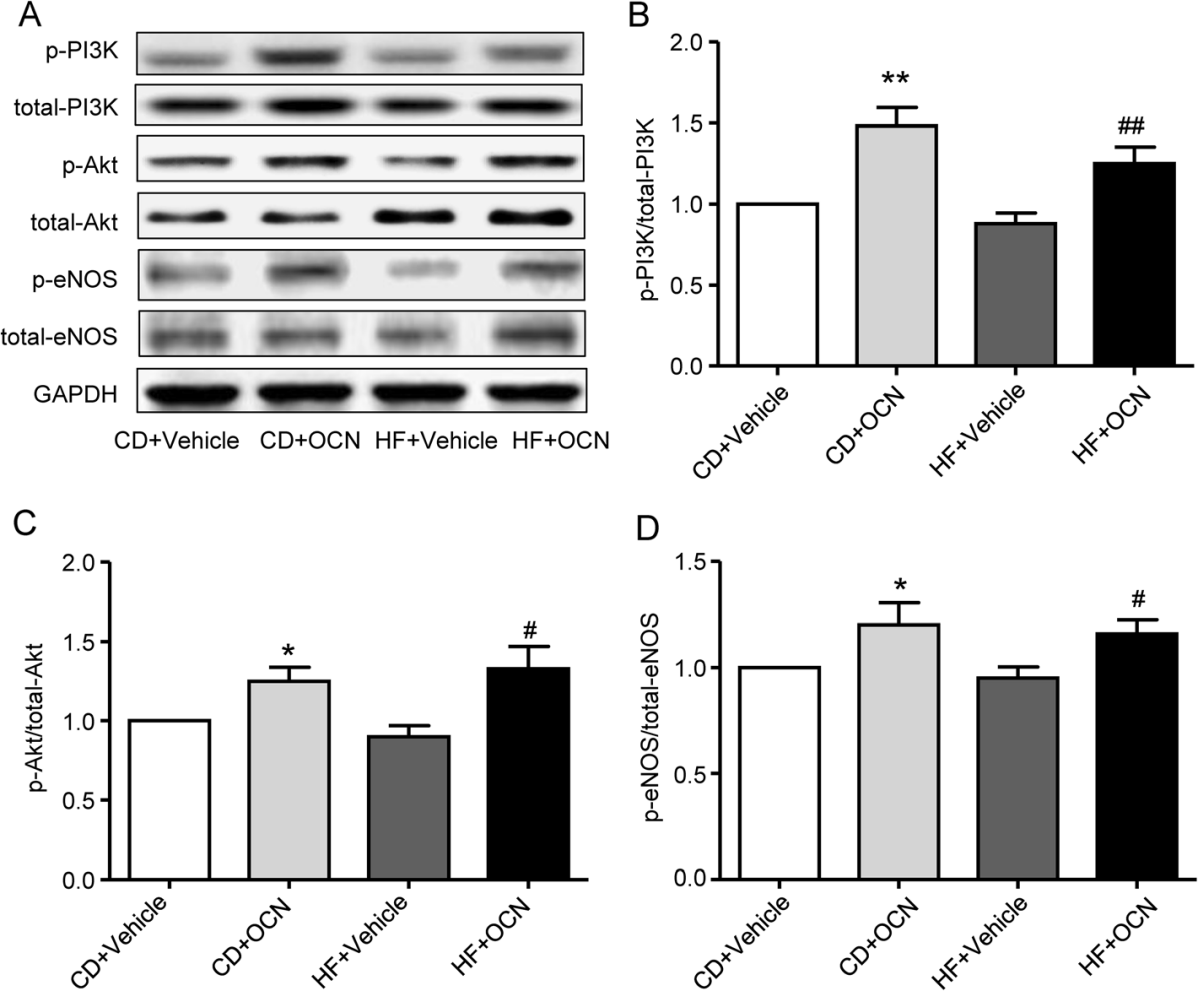
**Effect of OCN on PI3K, Akt and eNOS phosphorylation in descending thoracic aortic strips of ApoE-KO mice. (A)** Representative Western blot to show the expression of PI3K, phosphorylated PI3K, Akt, phosphorylated Akt, eNOS, phosphorylated eNOS. **(B)** Effect of OCN on PI3K phosphorylation. **(C)** Effect of OCN on Akt phosphorylation. **(D)** Effect of OCN on eNOS phosphorylation. Values represent mean ± SEM, 6 to 8 mice per group were analyzed. *P < 0.05, **P < 0.01, OCN-treated mice vs. vehicle-treated mice in chow diet group; ^#^*P* < 0.05, ^##^*P* < 0.01, OCN-treated mice vs. vehicle-treated mice in HFD group. HF, high fat diet; CD, chow diet; OCN, osteocalcin.

To investigate the mechanism of modulating vascular function in ApoE-KO mice treated with OCN, total and phosphorylated PI3K, Akt and eNOS expression were detected. As shown in Figure 5, OCN treatment significantly increased phosphorylation of PI3K, Akt and eNOS in both chow diet group and HFD group (all *P* < 0.05).

### OCN increases eNOS levels in HUVECs through activation of the Akt/eNOS pathway

**Figure 6:**
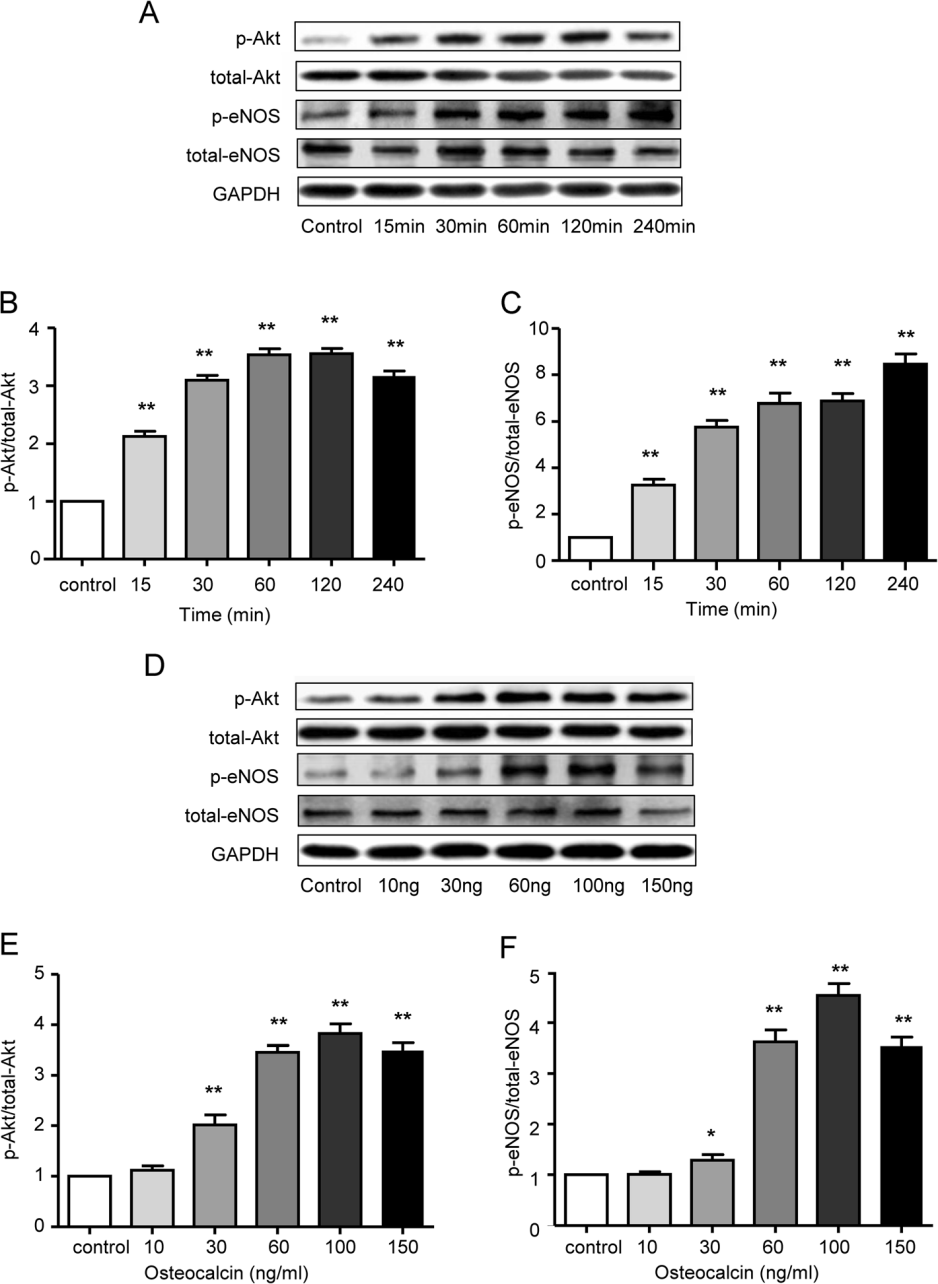
**Effect of OCN on the phosphorylation of Akt and eNOS in cultured HUVECs. (A)** HUVECs incubated with 100 ng/mL OCN or vehicle for the indicated times. **(B)** The effects of OCN on Akt phosphorylation. **(C)** The effects of OCN on eNOS phosphorylation. **(D)** HUVECs incubated with the indicated concentrations of OCN or vehicle for 1 h. **(E)** The effects of OCN on Akt phosphorylation. **(F)** The effects of OCN on eNOS phosphorylation. Mean ± SEM of four independent experiments are plotted. *P < 0.05, **P < 0.01 vs. control group. OCN, osteocalcin.

To determine the effect of OCN on Akt/eNOS pathway *in vitro*, the phosphorylation of Akt and eNOS was examined in the OCN-treated and untreated HUVECs. Incubation of the HUVECs with OCN (100 ng/ml) significantly increased Akt phosphorylation and eNOS phosphorylation in a time dependent manner (Figure 6A-C). Concerning the dose-related situation, incubation of HUVECs for 1 h with various concentrations of OCN demonstrated that Akt phosphorylation and eNOS phosphorylation were gradually increased after treatment with 30, 60, 100, 150 ng/ml of OCN (Figure 6D-F). Thus, OCN may activate Akt/eNOS pathway in HUVECs.

### The beneficial effect of OCN on EDR in ApoE-KO mice requires the activation of the PI3K/Akt signaling pathway

**Figure 7:**
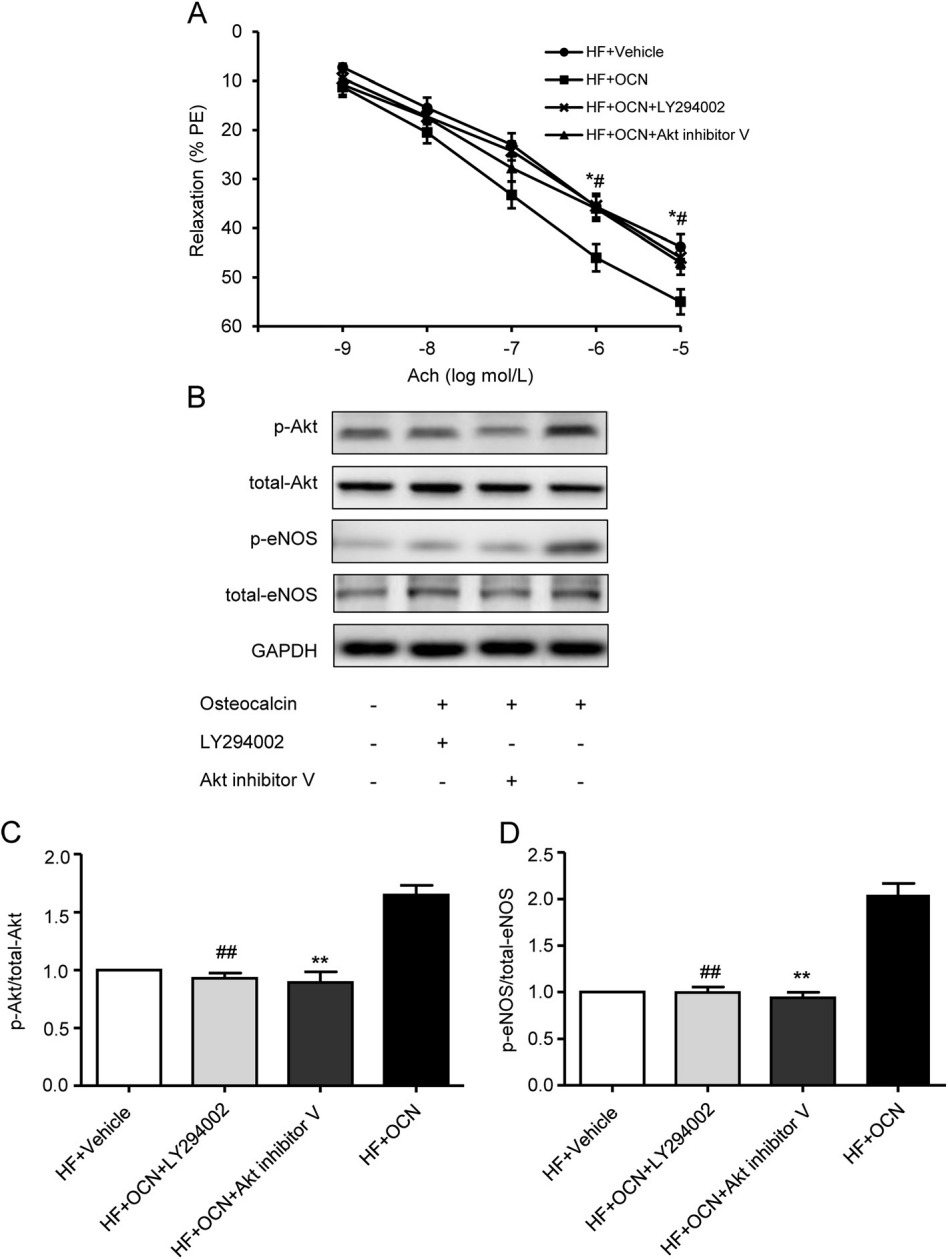
**Effect of OCN on ACh-stimulated EDR and PI3K/Akt signaling pathway in descending thoracic aortic strips of ApoE-KO mice fed with HFD. (A)** Beneficial effect of OCN treatment for 24 h on EDR. **(B)** OCN-mediated changes in phosphorylation of eNOS and Akt in the presence of PI3K inhibitor LY294002 or Akt inhibitor V. **(C)** The effects of OCN, LY294002 and Akt inhibitor V on Akt phosphorylation. **(D)** The effects of OCN, LY294002 and Akt inhibitor V on eNOS phosphorylation. Values represent mean ± SEM, n = 5-6. ^#^*P <* 0.05, ^##^*P <* 0.01, HF + OCN-treated + LY294002 vs. HF + OCN-treated group; *P < 0.05, **P < 0.01, HF + OCN-treated + Akt inhibitor V vs. HF + OCN-treated group. HF, high fat diet; EDR, endothelium-dependent relaxation; OCN, osteocalcin.

To investigate whether the PI3K/Akt pathway is required for the effect of OCN on EDR, PI3K inhibitor (LY294002) and Akt inhibitor V were applied in the medium for descending thoracic aortic strips. OCN treatment markedly improved the HFD-related impairment of EDR in descending thoracic aortic strips from ApoE-KO mice (Figure 7A). Coincubation with LY294002 (10 μmol/L) or Akt inhibitor V (5 μmol/L) could antagonized this beneficial effect, as well as inhibited the OCN treatment-related increases in eNOS phosphorylation at Ser1177 and Akt phosphorylation at Ser473 (Figure 7B-D). Taken together, these observations suggested that the mechanism underlying OCN’s protective effect on vascular function requires the activation of the PI3K/Akt signaling pathway.

## Discussion

This study has demonstrated that daily injections of OCN produced significant effects on glucose and lipid metabolism, as well as improving insulin sensitivity, in ApoE-KO mice, all of which represent risk factors of cardiovascular disease. Moreover, vascular EDR was significantly improved in thoracic aorta specimens from the OCN-treated ApoE-KO mice fed a high fat diet. Following incubation of the HUVECs or thoracic aortic strips with OCN, eNOS phosphorylation was significantly increased and HFD-related impairment of EDR was attenuated. It was determined that the protective effect of OCN was mediated, at least in part, by the activation of PI3K/Akt signaling pathway, which was consistent with the research of Jung et al. [15]. These results suggest that OCN plays an important role in modulating endothelial function *in vivo*.

Previous studies have shown that, in accord with the present study, the osteoblast-derived protein, OCN, affects lipid and glucose metabolic regulation in mice. In the current study of ApoE-KO mice, the daily injections of OCN induced decreases in FBG and serum level of lipids, and an increase in insulin secretion regardless of diet composition. Although the OCN injections had no significant effects on glucose tolerance and insulin sensitivity in the ApoE-KO mice on a chow diet, they did improve glucose tolerance and insulin sensitivity in ApoE-KO mice on a HFD. This suggests that intermittent injections of OCN have a more profound effect in mice with already altered insulin sensitivity [19]. Systemic metabolic abnormalities, such as dyslipidemia, hyperglycemia and insulin resistance, are important risk factors of cardiovascular disease [20,21]. Hypercholesterolemia is one of the characteristics of ApoE-KO mice [22]. Substantial clinical and experimental evidence has suggested that both hyperglycemia and dyslipidemia contribute to endothelial cell dysfunction. Hyperglycemia causes an accelerated formation of advanced glycation end-products and mitochondrial overproduction of reactive oxygen species. Dyslipidemia also strongly and directly enhances monocyte adhesion to endothelium. Both alterations can result in vascular injury and endothelial damage. Given that OCN can ameliorate dyslipidemia and impaired glucose metabolism, it may well constitute a protective factor for vascular disease. TNF-α, IL-1α and IL-12 are known as pathogenic factors during the development of atherosclerosis. Our results suggested that OCN may also play a role in alleviating chronic inflammation in ApoE-KO mice. In addition, several clinical studies have reported a significantly inverse correlation between OCN and blood pressure [23,24]. In the present study, a significant vascular protective effect of OCN, through its lowering of mean BP and diastolic BP, was found in ApoE-KO mice fed the HFD.

The endothelium plays an important role in maintaining vascular homeostasis by synthesizing and releasing several vasodilators, including NO and endothelium-derived hyperpolarizing factor (EDHF) [25]. In mouse aorta, NO is the sole endothelial-derived mediator [26]. Substantial clinical and experimental evidence suggests that endothelial dysfunction is an early marker for the initiation and progression of atherosclerosis [27]. ApoE-KO mice, as in the present study, are one of the most widely used animal models of atherosclerosis [28-30]. Endothelial dysfunction has been demonstrated in the aorta of ApoE-KO mice fed a western diet and is characterized by impaired ACh-induced endothelium-mediated aortic vasodilation. In accord with previous studies, our research found a reduced EDR to ACh in the aorta of ApoE-KO mice fed with HFD; this result serves as an important indicator of vascular dysfunction. Furthermore, an increased EDR to ACh was demonstrated in the OCN-treated HFD group. By contrast, the phenomenon was not observed in mice fed with chow diet. Previous studies suggested that the expression of total Akt significantly increased in HFD status, which may indicate a compensatory or adaptive mechanism in obese mice [31-33]. This might explain why OCN had a more significant effect on EDR in mice fed with HFD through the regulation of the Akt/eNOS-dependent pathway. Application of L-NAME abolished EDR in all of the ApoE-KO mice, regardless of diet composition, similar to the findings of other researchers [34]. However, OCN did not appear to affect the ability of VSMCs to respond to NO. Thus, OCN may play a positive role in endothelial vasodilatory function and this beneficial effect may result from, at least partially, its favorable modulation of glucose and lipid metabolism by way of its protective effect against endothelial dysfunction otherwise induced by glucose and lipid metabolism disorders.

This study also sought to determine the possible mechanism that underlies the relationship between OCN and vascular EDR. Altered eNOS-NO signaling is a common feature observed in animal models exhibiting impaired endothelial function [35-38]. Phosphorylation at Ser1177 is necessary for the maximal activation of eNOS and results in optimal NO production [39]. In the present study, expression of P-eNOS and total eNOS was increased in descending thoracic aortic strips of OCN-treated ApoE-KO mice fed with HFD, compared with the vehicle-treated ApoE-KO mice fed with HFD. In addition, similar results were found in OCN-treated HUVECs, and this *in vitro* finding agreed with those of a previous study by this group [15]. Collectively, these results suggest that eNOS-NO signaling may contribute to the OCN-mediated modulation of vasorelaxation in ApoE-KO mice.

In the endothelium, PI3K/Akt signaling mostly acts as a positive regulator of endothelial NO synthase, which generates NO through the NADPH-dependent oxidation of L-arginine [40,41]. Regarding the regulation of eNOS by PI3K/Akt signaling, it has previously been shown that activated Akt phosphorylates eNOS on Ser1177, enhancing both basal and stimulated eNOS enzyme activity, and thereby NO release [42,43]. In contrast, loss of the Akt1 subtype in the vessel wall is associated with reduced eNOS phosphorylation [44,45]. In the present study, phosphorylation of both Akt and eNOS was significantly increased in HUVECs in response to OCN treatment. Furthermore, a beneficial effect of OCN was observed using an *ex vivo* organ culture of isolated mouse aortic strips, which was attenuated upon coincubation with a PI3K or Akt inhibitor. These results suggest that OCN may protect vascular endothelial cell from impairment, in part, by activating the PI3K/Akt signaling pathway.

## Conclusions

In summary, the current study supported that daily injections of OCN is likely to reduce the risk of cardiovascular disease, as it improves glucose and lipid metabolism. Furthermore, a protective effect of OCN on vascular EDR was demonstrated. Such beneficial activities of OCN*,* which lead to an increase of eNOS phosphorylation, required the activation of the PI3K/Akt/eNOS signaling pathway. These novel findings may help to enhance the prospective of OCN in combating vascular dysfunction in atherosclerosis.

## Abbreviations

ApoE–KO: Apolipoprotein E-deficient; Ach: Acetylcholine; BP: Blood pressure; EDR: Endothelium-dependent relaxation; Emax: Maximal relaxation; FBG: Fasting blood glucose; GTT: Glucose tolerance tests; HDL-C: High-density lipoprotein cholesterol; IPGTT: Intraperitoneal glucose tolerance tests; ITT: Insulin tolerance tests; HUVECs: Human umbilical vein endothelial cells; HFD: High fat diet; L-NAME: *N*^G^-nitro-L-arginine methyl ester; LDL-C: Low-density lipoprotein cholesterol; OCN: Osteocalcin; PE: Phenylephrine; SDS-PAGE: Sodium dodecyl sulfate polyacrylamide gel electrophoresis; SEM: Standard error of the mean; SNP: Sodium nitroprusside; TG: Triglycerides; TC: Total cholesterol; VSMC: Vascular smooth muscle cell.

## Competing interests

The author(s) declare that they have no competing interests.

## Authors’ contributions

YB and WJ designed the study. JD, MZ, and MN carried out the experiments. JD, XM and QF analyzed data and wrote the draft. HL, YB, and WJ revised the paper and contributed to the discussion. All authors read and approved the final manuscript.

## Supplementary Material

Additional file 1: Figure S1 Representative photographs of Oil-Red-O staining of aortic arches in ApoE-KO mice. All of the ApoE-KO mice have developed atherosclerosis after the 12th week of experimental intervention. HF, high fat diet; CD, chow diet; OCN, osteocalcin. Click here for file
