# Supplementary figures and images for: Osteocalcin attenuates high fat diet-induced impairment of endothelium-dependent relaxation through Akt/eNOS-dependent pathway
Source: Cardiovasc Diabetol. 2014 Apr 7;13:74. doi: 10.1186/1475-2840-13-74 (PMC4233640; doi:10.1186/1475-2840-13-74)

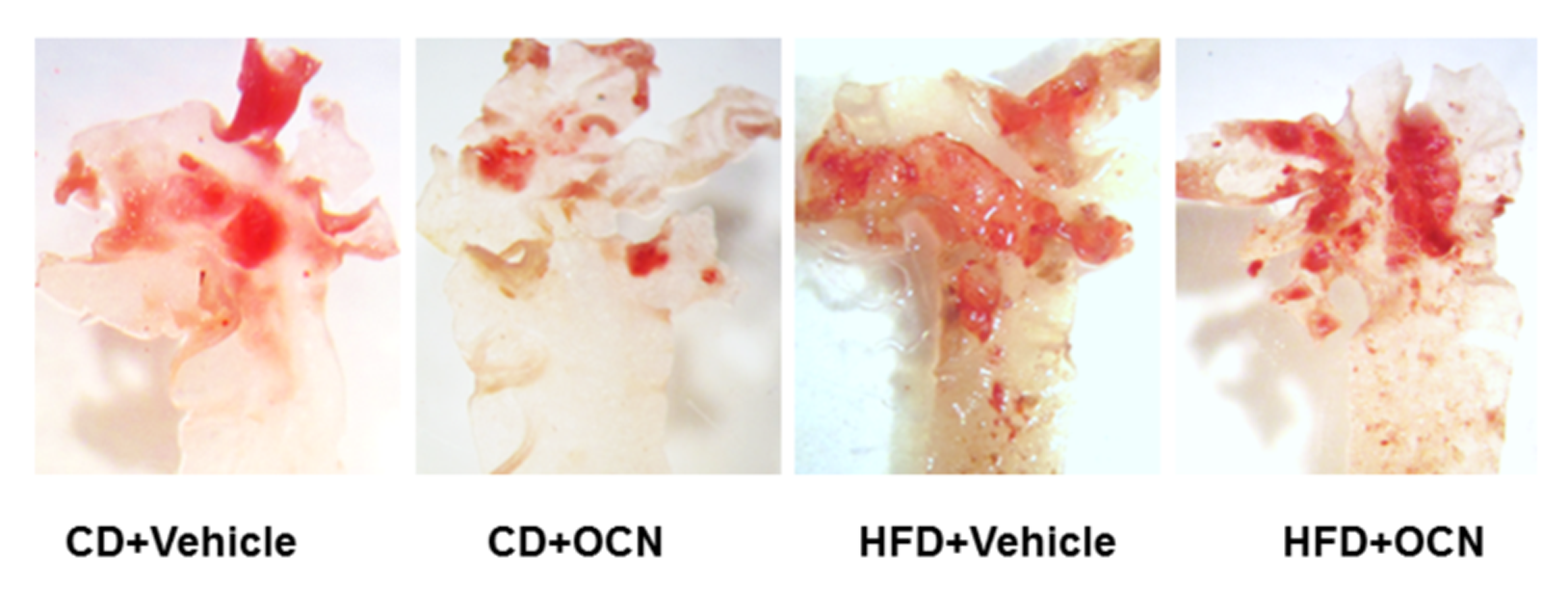

Supplement: Additional file 1: Figure S1 — Representative photographs of Oil-Red-O staining of aortic arches in ApoE-KO mice. All of the ApoE-KO mice have developed atherosclerosis after the 12th week of experimental intervention. HF, high fat diet; CD, chow diet; OCN, osteocalcin. [file 1475-2840-13-74-S1.tiff]
